# Early life acute infections and risk for cow's milk protein allergy or atopic dermatitis at 6 months of age in high risk for allergy infants

**DOI:** 10.3389/fped.2024.1424331

**Published:** 2024-12-16

**Authors:** Rouzha Pancheva, Zoi Illiodromiti, George Moschonis, Eva Kontopodi, Eleni Karapati, Nicolaos Nicolaou, Eva Karaglani, Mikaela Sekkidou, Simoneta Popova, Nataliya Usheva, Miglena Marinova, Paraskevi Xepapadaki, Olympia Sardeli, Anastasia Kapetanaki, Nicoletta Iacovidou, Theodora Boutsikou, Evangelia Papathoma, Yannis Manios

**Affiliations:** ^1^ Department of Hygiene and Epidemiology, Faculty of Public Health, “Prof. Dr. Paraskev Stoyanov” Medical University—Varna, Varna, Bulgaria; ^2^ Research Group NutriLect, Department of Neuroscience, Research Institute, Medical University “Prof. Dr. Paraskev Stoyanov”—Varna, Varna, Bulgaria; ^3^ Neonatal Department, Medical School, Aretaieio Hospital, National and Kapodistrian University of Athens, Athens, Greece; ^4^ Discipline of Food, Nutrition and Dietetics, Department of Sport, Exercise and Nutrition Sciences, School Allied Health, Human Services and Sport, La Trobe University, Melbourne, VIC, Australia; ^5^ FrieslandCampina, Amersfoort, Netherlands; ^6^ University of Nicosia Medical School, Nicosia, Cyprus; ^7^ Asthma and Allergy Center, Limassol, Cyprus; ^8^ Department of Nutrition & Dietetics, School of Health Science & Education, Harokopio University, Athens, Greece; ^9^ Department of Social Medicine and Healthcare Organization, Faculty of Public Health, “Prof. Dr. Paraskev Stoyanov” Medical University—Varna, Varna, Bulgaria; ^10^ Allergy Department, 2nd Pediatric Clinic, National and Kapodistrian University of Athens, Athens, Greece; ^11^ Third Department of Pediatrics, National and Kapodistrian University of Athens, Attikon General University Hospital, Athens, Greece; ^12^ Neonatology Department, Helena Venizelou Hospital, Athens, Greece; ^13^ Neonatal Department, National and Kapodistrian University of Athens, Aretaieio Hospital, Athens, Greece; ^14^ Neonatal Intensive Care Unit, Alexandra University and State Maternity Hospital, Athens, Greece; ^15^ Institute of Agri-Food and Life Sciences, Hellenic Mediterranean University Research Centre, Heraklion, Greece

**Keywords:** early life infections, cow's milk protein allergy, atopic dermatitis, infant feeding, allergy prevention, allergy reduction trial

## Abstract

**Background:**

Early life infections (ELIs), encompassing both viral and bacterial types, occur within the first six months of life. Influenced by genetic host factors and environmental conditions, the relationship between ELIs and subsequent allergic manifestations, particularly cow's milk protein allergy (CMPA) and atopic dermatitis (AD), is complex and not fully understood.

**Objective:**

The aim of the current study was to examine the potential interplay between nutrition, infections, and allergic manifestations in the first six months of life in infants with a family history of allergies, who were either exclusively breastfed (EBF) or fed a combination of breast milk and standard (SF) or partially hydrolyzed infant formula (pHF).

**Methods:**

The Allergy Reduction Trial (ART) is a multicenter, randomized controlled trial involving 551 participants. From birth, these participants were divided into three groups: Exclusive Breastfeeding (EBF), Partially Hydrolyzed Formula (pHF), and Standard Formula (SF). ELIs, defined as viral and bacterial infections occurring during the first 6 months, and outcomes (AD, CMPA) were recorded through questionnaires (i.e., SCORAD and CоMiSS) and clinical assessments.

**Results:**

The relative risk (RR) for CMPA in infants with ELIs was 0.20 (95% CI: 0.07–0.58), highlighting a protective effect of ELIs against CMPA development. Notably, the incidence of CMPA was significantly lower in infants who experienced ELIs compared to those without (3% vs. 13.4%, *p* = 0.001), with no cases of CMPA observed at 6 months in exclusively breastfed (EBF) infants with ELIs. For AD, a trend was observed where the incidence was lower in infants with ELIs who were fed with pHF at 6.5%, compared to those fed with SF at 18.2% (*p* = 0.092), suggesting a potential protective effect of ELIs in the pHF group against AD development.

**Conclusion:**

The study highlights a potential protective role of ELIs in reducing the risk of CMPA, particularly in EBF infants. Furthermore, it suggests a trend towards lower AD incidence in infants fed with pHF, highlighting the complex interplay between early microbial exposures, feeding practices, and immune development. Further research is warranted to unravel this challenging relationship and appropriately inform early life allergy prevention strategies

## Introduction

Early life infections (ELIs), encompassing both viral and bacterial types, occur within the first six months of life. These infections are influenced by a blend of genetic host factors and various environmental factors (1). Notable among these are ceasarean delivery, antibiotic misuse, early nutrition, prematurity, low birth weight, early daycare attendance, and crowding (2). Besides these factors, ELIs have also been linked to allergic conditions, which are further affected by variables such as allergic heredity, late exposure to allergens, and tobacco exposure, either *in utero* or from the environment in (3).

A critical factor in the development of an infant's immune system is the modulation of the gastrointestinal microbiome, especially between the ages of 3 and 6 months (4). This microbiome is shaped by various influences, including delivery mode, diet, antibiotic exposure, the immediate environment exposure, and maternal BMI. These factors not only determine the microbiome's diversity but also the abundance of specific microbial populations (4). According to the hygiene hypothesis, early antigen exposure, not necessarily pathogenic, is essential for developing a microbiome that trains the immune system to tolerate various stimuli, including allergens later in life (5). This training potentially involves a balance between Th1 and Th2 immune responses, influenced by early exposure levels to infections and the interaction between gut epithelial cells, mucosal immune cells, and microbiota. This interaction is crucial for maintaining an intact intestinal epithelial barrier, thus preventing the permeation of pathogens, toxins, and allergens in the body that could trigger the immune system and lead to chronic inflammation (leaky gut hypothesis) (6).

However, the relationship between ELIs and subsequent allergic manifestations is complex and not fully understood. Infants with a predisposition to atopy have delayed maturation of Th1 responses in childhood, which puts them at increased infection risks during the first years of life (7). Certain infections, like early life rotavirus, recurrent gastroenteritis, and lower airway infections caused by Respiratory Syncytial Virus and Rhinoviruses, have been identified as risk factors for conditions such as wheezing, asthma, and allergic predisposition (8–11). Neonatal and infant bacterial infections and associated antibiotic use also seem to predispose infants to allergies by altering the gut microbiome (12, 13). Conversely, some studies suggest that respiratory or gastrointestinal infections do not significantly impact allergic predisposition (14). In support of the hygiene hypothesis, vaccinations and infections such as Bacillus Calmette-Guérin (BCG), Mycobacterium tuberculosis, Hepatitis A, and measles have been observed to reduce allergic incidences later in life (5).

While there is comprehensive evidence for the multiple benefits of breastfeeding, such as supporting the gut barrier, offering anti-inflammatory molecules, and encouraging a beneficial microbiota largely attributed to human milk oligosaccharides, its role in reducing the risk of cow's milk protein allergy (CMPA) is not conclusively established. Studies have reported varying outcomes, ranging from a protective effect to no effect or even a potential predisposing effect towards CMPA (15, 16). Breastfeeding also offers protection against respiratory and gastrointestinal infections through both active and passive immunity mechanisms, including components like lysozyme, interleukins, fibronectin, and the lactoperoxidase system, along with immunoglobulins (17). Interestingly, a study showed that exclusive breastfeeding for more than 4 months is associated with a lower incidence of CMPA than other feeding regimens (18), while another (19) showed that early exposure to cow's milk protein is protective against IgE-mediated cow's milk protein allergy.

Alternative feeding methods for non-exclusively breastfed infants, including extensively and partially hydrolyzed infant formulas, have shown potential in reducing the risk of atopic dermatitis (AD) and possibly other allergic conditions in allergy-prone infants (20, 21). Some evidence suggests that early exposure to allergens may prevent the onset of allergy (22–25) while introducing intact cow's milk formula between one and three months might reduce CMPA incidence at six months (24). According to the European Society for Paediatric Gastroenterology Hepatology and Nutrition (ESPGHAN), it is still unclear whether avoiding regular consumption of cow's milk-based formula during early life reduces or increases the risk of CMPA in infants (25). The efficacy of partially hydrolyzed formulas in reducing the risk of allergy development is debated, though studies like GINI (20), Halken et al. (26), and the first results of the A.R.T. study on allergy prevention (27), reported a possible beneficial effect. This protection may arise from exposing the gut-associated lymphoid tissue (GALT) to smaller peptides, potentially fostering food tolerance without sensitization, thus reducing intestinal permeability and inflammatory markers (21).

Given these insights, the aim of the current study was to examine the potential interplay between nutrition, infections, and allergic manifestations in the first six months of life in infants with a family history of allergies, who were either exclusively breastfed or exclusively breastfed or formula fed with a standard or partially hydrolysed infant formula (exclusively or in combination with breast milk).

## Methods

### Study design and participants

The current manuscript is a secondary data analysis of A.R.T. study, which was a multicenter, double-blinded, parallel-group, randomized controlled trial. The primary objective of A.R.T. was to evaluate the incidence of CMPA and AD in healthy term infants at high risk of allergy (27, 28). For the present analysis, adverse events (AEs) which were reported during the bi-monthly follow-up visits, were used to identify ELIs. Participating infants, all of whom were exposed exclusively to breast milk (colostrum) within the first 24 h post-delivery, were then randomly allocated either to the exclusive breastfeeding (EBF) group or, if not continuing with exclusive breastfeeding, to one of two formula groups: a partially hydrolyzed whey-based formula (pHF) or a standard intact protein cow's milk formula (SF). These formulas, provided by FrieslandCampina, were similar in macronutrients, differing primarily in protein structure (partially hydrolyzed vs. intact). Infants in the mixed-feeding group were required to consume at least 40 ml/kg of body weight per day of formula at 1 month and 60 ml/Kg of body weight at 2 months. Mixed-feeding allocation was permitted until 10 weeks of age.

ELIs were monitored through adverse event reports. Diagnosis of CMPA was made by pediatricians and/or paediatric allergists following the A.R.T. protocol. The SCORAD (Scoring of Atopic Dermatitis) (29) tool aided in assessing the severity of AD, and CoMiSS (Cow's Milk-related Symptom Score) (30) in identifying infants with suggestive CMPA and other allergic manifestations.

The A.R.T. study was conducted in Bulgaria, Cyprus, and Greece from 2017 to 2019 and is registered in the Netherlands Trial Registry [Trial NL6120 (NTR6259)]. The study protocol, information letter to the parents/legal guardians, and written informed consent form were reviewed and approved by the appropriate independent ethics committees in each center: Bulgaria: Research Ethics Committee of Medical University of Varna; Cyprus: Cyprus National Bioethics Committee; Greece: (a) Research Ethics Committee of Alexandra Hospital, (b) Research Ethics Committee of Areteio Hospital, (c) Research Ethics Committee of Attikon Hospital, (d) Research Ethics Committee of Helena Hospital (27).

### Recruitment procedures and inclusion criteria

The recruitment procedures and detailed methodology of A.R.T. study have been described previously (27). Briefly, potential participants were identified through interviews with families at maternity clinics and those with a family history of allergy were offered information about the A.R.T. study. Written consent to participate was obtained shortly after delivery, following detailed discussions and the fulfillment of inclusion criteria.

### Follow-up evaluation and compliance

Infants were monitored bi-monthly for the first six months, with additional evaluations as needed for allergy signs. At each visit, clinical assessments for CMPA and AD symptoms were conducted using the SCORAD and CoMiSS, along with a questionnaire for IgE- and non-IgE-mediated food allergy symptoms.

Formula intake and breastfeeding duration was recorded using a 7-day diary prior to the 1st, 2nd, 4th, and 6th month visits. Breastmilk intake in mixed-fed infants was estimated using a specific equation (31). For breastfed infants, no dietary restrictions were placed on mothers, and exclusive breastfeeding was maintained at least until 4 months.

### Definition of study outcomes

#### Cow's milk protein allergy (CMPA)

CMPA diagnosis followed EAACI and USA guidelines, using skin prick testing, CMP elimination diets, oral food challenges (OFC), and clinical tools like SCORAD and CoMiSS. Full details are described in (27).

#### Atopic dermatitis (AD)

AD diagnosis relied on clinical assessment and severity scoring using SCORAD and CoMiSS, with criteria outlined in (27).

ELIs were identified from AEs reports, where information on fever, acute diarrhea, constipation, coughing, accidents, infections, and other AEs were recorded. These AEs were meticulously documented, classified as infection or non-infection, and assessed for potential relation to the study product. Acute infection diagnoses were primarily made in primary care and recorded in patient files. Detailed information about each AE episode was collected at study visits, with additional follow-up via phone calls as needed.

Serious adverse events (SAEs) were defined by criteria including death, life-threatening situations, hospitalization, disability, or other significant health impacts. These SAEs were thoroughly documented, and the study center was notified within 24 h of occurrence. Hospital admissions and diagnoses for all SAEs, including acute infections, were recorded in participants' records.

### Statistical analysis

The sample size calculation of A.R.T. study has been described in detail elsewhere (27). All statistical analyses of the present study were conducted using the SPSS statistical software for Windows (IBM, version 28.0; IBM, Armonk, NY, USA). The normality of the distribution of continuous variables was tested by the Kolmogorov–Smirnov test. As all continues variables examined were normally distributed, they are presented as means and standard deviations (SD). Categorical variables are presented as frequencies (*n*) and percentages (%).

Both intention–to-treat (ITT) and per protocol (PP) statistical analyses were conducted, with the results from the latter been summarized in Supplementary Tables S1–S3. Between-group differences of continuous variables were tested using the one-way Analysis of Variance (ANOVA), while between-group differences of categorical variables were examined using the chi-squared (*χ*^2^) or the Fisher exact test, wherever appropriate. The incidence rates (%), relative risk (RR) and 95% confidence interval of RR (95% CI) for the occurrence of AD and CMPA at six months of life, were calculated based on the occurrence or not of ELIs, for the total sample and by study group. All logistic regression models performed to calculate RRs were adjusted for those variables that were found to be significantly different among the three study groups at baseline (i.e., country of infant's birth, the mode of the child's delivery, mother's educational level and urban residence), but also family history of allergies. All reported *p*-values were two-tailed, and the level of statistical significance was set at *p* < 0.05.

## Results

### Study population characteristics

A detailed summary of the demographic and clinical characteristics of the study population, including 220 infants in the EBF group, 160 infants in the pHF group, and 171 infants in the SF group is available in another article (27).

Table 1 provides an overview of the frequency of occurrence of certain AEs and SAEs classified as infections in each one of the three study groups (i.e., EBF, pHF, and SF groups) and the total study sample.

**Table 1:** Adverse events and serious adverse events in different groups (ITT analyses).

	EBF (*N* = 220)	pHF group (*N* = 160)	SF group (*N* = 171)	Total sample (*N* = 551)
AE-fever >1day, *n* (%)	5 (2.3%)	5 (3.1%)	5 (2.9%)	15 (2.7%)
AE-acute diarrhoea > 1day, *n* (%)	9 (4.1%)	6 (3.8%)	9 (5.3%)	24 (4.4%)
AE-respiratory infection > 1day, *n* (%)	5 (2.3%)	5 (3.1%)	6 (3.5%)	16 (2.9%)
AE-other infections, *n* (%)	20 (9.1%)	22 (13.8%)	23 (13.5%)	65 (11.8%)
SAE-classified as infection, *n* (%)	6 (2.7%)	10 (6.3%)	11 (6.4%)	27 (4.9%)

EBF, exclusive breastfeeding; pHF, partially hydrolysed formula; SF, Standard formula; ITT, Intention-to-treat; AE, adverse events; SAE, serious adverse events; *N*, Number of study participants; *n*, number of non-missing observations.

According to the data presented in Table 2, the majority of infants in the total sample were recruited from Bulgaria (37.4%), were males (54.8%), were reported having no ELIs (75.5%), had a mean birth weight of 3,286.1 (SD: 423.6) g and a mean length at birth of 49.9 (SD: 1.9) cm. Furthermore, the percentages of infants that were delivered via caesarean section were higher in the pHF and SF groups, compared to the EBF group (66.3% and 62% vs. 45.9% respectively; *p* < 0.001) Regarding maternal characteristics, the mean age of mothers was 31.9 (SD: 5.1) years, approximately two thirds (65.5%) of mothers had more than 14 years of education, while 9.1% reported smoking during their pregnancy. As for the characteristics of recruited families, the vast majority (89.1%) lived in urban areas, while only 19.8% had a pet that they kept indoors at home. As far as family medical history of allergies was concerned, rhinitis was the most prevalent allergy (53.7%), followed by food allergy (30.3%), AD (29.9%), allergic asthma (27.2%) and urticaria (14.9%). Regarding differences between study groups, in Greece, a higher proportion of infants were exclusively breastfed compared to infants fed with pHF or SF (i.e., 51.8% in EBF group vs. 18.1% in pHF group vs. 16.4% in SF group; *p* < 0.001), while the opposite was observed in Bulgaria (i.e., 21.8% in EBF group vs. 47.5% in pHF group vs. 48.0% in SF group; *p* < 0.001). Furthermore, the percentage of mothers with more than 14 years of education was higher in the EBF compared to the SF group (72.7% vs. 59.1%, *p* = 0.012). A similar finding was observed for the percentage of families living in urban areas, which was also higher in the EBF compared to the SF group (i.e., 92.3% vs. 82.9%, *p* = 0.008). No other significant differences were observed between study groups.

**Table 2:** Descriptive characteristics of study participants allocated to the three study groups at baseline (ITT analyses).

	Study groups				
	EBF group (*N* = 220)	pHF group (*N* = 160)	SF group (*N* = 171)	*p*-value	Total sample (*N* = 551)
Infant's characteristics					
Country of infant's birth					
Bulgaria, *n* (%)	48 (21.8)^a^^,^^b^	76 (47.5)^a^	82 (48.0)^b^	**<0** **.** **001**	206 (37.4)
Cyprus, *n* (%)	58 (26.4)	55 (34.4)	61 (35.7)		174 (31.6)
Greece, *n* (%)	114 (51.8)^a.b^	29 (18.1)^a^	28 (16.4)^b^		171 (31.0)
Mode of delivery					
Caesarean section (%)	101 (45.9)^a^^,^^b^	106 (66.3)^a^	106 (62.0)^b^	**<0** **.** **001**	313 (56.8)
Birth weight, g, mean (SD)	3,303.6 (392.3)	3,270.5 (433.6)	3,278.1 (453.7)	0.722	3,286.1 (423.6)
Length at baseline, cm, mean (SD)	50.1 (2.0)	49.7 (1.8)	49.8 (1.8)	0.075	49.9 (1.9)
Gender, males, *n* (%)	116 (52.7)	93 (58.1)	93 (54.4)	0.575	302 (54.8)
Mother's characteristics					
Age, years, mean (SD)	32.5 (4.9)	31.7 (5.1)	31.3 (5.1)	0.062	31.9 (5.1)
Educational level					
≤ 14 years, *n* (%)	60 (27.3)^b^	60 (37.5)	70 (40.9)^b^	**0** **.** **012**	190 (34.5)
>14 years, *n* (%)	160 (72.7)^b^	100 (62.5)	101 (59.1)^b^		361 (65.5)
Mother smoking during pregnancy, *n* (%)	13 (5.9)	15 (9.4)	22 (12.9)	0.059	50 (9.1)
Family characteristics					
Urban residence, *n* (%)	203 (92.3)^b^	146 (91.3)	141 (82.9)^b^	**0** **.** **008**	490 (89.1)
Presence of pets indoors at home, *n* (%)	39 (17.7)	32 (20.0)	38 (22.2)	0.207	109 (19.8)
Medical history					
Family history of:					
Allergic asthma, *n* (%)	66 (30.0)	42 (26.3)	42 (24.6)	0.462	150 (27.2)
Rhinitis, *n* (%)	130 (59.1)	81 (50.6)	85 (49.7)	0.118	296 (53.7)
Atopic dermatitis, *n* (%)	75 (34.1)	46 (28.7)	44 (25.7)	0.186	165 (29.9)
Urticaria, *n* (%)	32 (14.6)	22 (13.8)	28 (16.4)	0.789	82 (14.9)
Food allergy, *n* (%)	70 (31.8)	45 (28.1)	52 (30.4)	0.741	167 (30.3)
Occurrence of ELIs in infants					
No infections, *n* (%)	176 (80.0)	114 (71.3)	127 (74.3)	0.177	417 (75.7)
ELIs^†^, *n* (%)	44 (20.0)	46 (28.7)	44 (25.7)		134 (24.3)

^†^ ELIs, Any infection occurring early in life, i.e., within 6 months of life; EBF, exclusive breastfeeding; pHF, partially hydrolysed formula; SF, Standard formula; ITT, Intention-to-treat; *N*, Number of study participants; *n*, number of non-missing observations; SD, Standard Deviation.

*P*-values for the comparison of categorical variables derived from the *χ*^2^ test or the Fisher exact test, whenever appropriate. *P*-values for the comparison of continuous variables derived from one-way ANOVA. All *p*-values in bold font indicate statistically significant differences between study groups.

^a,b^ Percentages sharing the same superscript letter within the same line are statistically significantly different between them, according to pairwise comparisons using the Bonferroni correction to account for type I error.

Percentages sharing the same superscript letter within the same line are statistically significantly different between them, according to pairwise comparisons using the Bonferroni correction to account for type I error.

### Incidence of CMPA, AD and effect of early life infections

The incidence rates of AD or CMPA at the age of 6 months, for infants in the total sample and by study group, with or without ELIs are presented in Table 3. According to these findings the incidence of CMPA was significantly lower in infants with ELIs compared to those with no ELIs (i.e., 3% vs. 13.4%, *p* = 0.001). Following stratification by study group, a similar finding was also observed for infants in the EBF group, since there were no infants developing CMPA at 6 months from those that were exposed to ELIs compared to those that were not (i.e., 0% vs. 13.8%; *p* = 0.009). Interestingly, when infants were further stratified based on the timing of ELIs, the incidence of AD within 6 months was lower in infants fed with pHF and for whom infections were reported after the first month of life, compared to those with infections occurring before the first month of life and those with no ELIs (5% vs. 50% vs. 14.3%, respectively). However, these differences were of borderline significance (*p* = 0.079) (Supplementary Table S6). Regarding infants fed with SF, the incidence of AD was similar between those with ELIs and those without, at 18.9% vs. 18.2%, respectively. For CMPA, the incidence was 15.7% in infants without ELIs compared to 6.8% in those with ELIs. These differences, however, were not statistically significant (*p* = 0.148), suggesting that ELIs did not markedly influence the risk of AD or CMPA at 6 months in infants fed with SF. Some differences of borderline significance were also observed when comparing the incidence of AD and CMPA in infants with ELIs between study groups. In this regard, the incidence of AD was lower in infants with ELIs who were fed with the pHF compared to those fed with SF (6.5% vs. 18.2%, *p* = 0.092) (Supplementary Table S8). Furthermore, the incidence of CMPA was lower in infants with ELIs in the EBF group compared to those in the SF group (0% vs. 6.8%, *p* = 0.080). No other significant differences were observed for the incidence of AD or CMPA.

**Table 3:** Incidence of atopic dermatitis (AD) or cow's milk protein allergy (CMPA) at the age of 6 months for infants with or no infections at early life, in the total sample and by study group (ITT analyses).

Total sample							
Atopic dermatitis (AD)	Timing of infections				Timing of infections		
	No infection, *n* (%, 95% CI)	ELIs^†^ *n* (%, 95% CI)	*p*-value	Cow's milk protein allergy (CMPA + susp)	No infection, *n* (%, 95% CI)	ELIs^†^ *n* (%, 95% CI)	*p*-value
No AD	347 (83.2%; 81.4%–85.0%)	117 (87.3%; 82.4%−92.2%)	0.258	No CMPA	265 (86.6%; 84.1%−89.2%)^a^	128 (97.0%; 94.5%−99.5%)^a^	**0** **.** **001**
AD	70 (16.8%; 15%−18.6%)	17 (12.7%; 7.8%−17.6%)		CMPA	41 (13.4%; 10.9%−15.9%)^a^	4 (3.0%; 0.5%−5.5%)^a^	
Total	417 (100.0%)	134 (100.0%)		Total	306 (100.0%)	132 (100.0%)	
Exclusive Breastfeeding (EBF)							
No AD	144 (81.8%; 79.2%−84.4%)	38 (86.4%; 77.3%−95.5%)	0.476	No CMPA	125 (82.6%; 79.0%−86.2%)	44 (100.0%; 100%−100%)	**0** **.** **009**
AD	32 (18.2%; 15.6%−20.8%)	6 (13.6%; 4.5%−22.7%)		CMPA	20 (13.8%; 10.5%−17.1%)	0 (0.0%; 0%−0%)	
Total	176 (100.0%)	44 (100.0%)		Total	145 (100.0%)	44 (100.0%)	
Partially hydrolysed formula (pHF)							
No AD	100 (87.7%; 84.5%−90.9%)	43 (93.5%; 87.5%−99.5%)	0.285	No CMPA	65 (90.3%; 85.2%−95.4%)	43 (97.2%; 93.0%−100%)	0.124
AD	14 (12.3%; 9.1%−15.5%)	3 (6.5%; 0.5%−12.5%)		CMPA	7 (9.7%; 4.6%−14.8%)	1 (2.3%; 0%−6.1%)	
Total	114 (100.0%)	46 (100.0%)		Total	72 (100.0%)	44 (100.0%)	
Standard formula (SF)							
No AD	103 (81.1%; 77.6%−84.5%)	36 (81.8%; 71.9%−91.7%)	0.916	No CMPA	75 (84.3%; 79.1%−89.6%)	41 (93.2%; 86.8%−99.6%)	0.148
AD	24 (18.9%; 15.4%−22.4%)	8 (18.2%; 8.4%−28.1%)		CMPA	14 (15.7%; 10.5%−21.0%)	3 (6.8%; 0.4%−13.2%)	
Total	127 (100.0%)	44 (100.0%)		Total	89 (100.0%)	44 (100.0%)	

95% CI, 95% Confidence Interval; ^†^ELIs, Any infection occurring early in life, i.e., before or after the 1st month of life; ITT, intention-to-treat.

*P*-values for the comparison of categorical variables derived from the *χ*^2^ test or the Fisher exact test, wherever appropriate. All *p*-values in bold font indicate statistically significant differences among study groups; Percentages sharing the same superscript letter within the same line are statistically significantly different between groups.

### Comparative analysis of feeding groups

Table 4 summarizes the RR (95% CIs) for the occurrence of AD or CMPA at the age of 6 months for infants in the total sample and by study group, based on the presence or not of ELIs. These findings highlight a lower RR for CMPA of 0.20 (95% CI: 0.07–0.58) for infants with ELIs compared to those with no infections. Although the presence of ELIs in infants in the pHF group resulted to almost half the risk for developing AD and CMPA compared to the SF group, the relevant RRs (0.62 and 0.23 in pHF vs. 0.96 and 0.38 in SF respectively) were not of statistical significance. No other statistically significant RRs were observed either for AD or CMPA, while the RR for infants in the EBF group could not be calculated as there were no exclusively breastfed infants diagnosed with CMPA within 6 months, for whom an early life infection was also reported.

**Table 4:** Relative risks for atopic dermatitis (AD) or cow's milk protein allergy (CMPA) at the age of 6 months based on the presences or not of early life infection, in the total sample and by study group (ITT analyses).

	Atopic dermatitis (AD)	Cow's milk protein allergy (CMPA + susp)
Total sample		
Timing of infections	**RR (95% CI)**	**RR (95% CI)**
No infection	1.00	1.00
ELIs^†^	0.77 (0.43–1.39)	**0.20 (0.07–0.58)**
Exclusive Breastfeeding (EBF)		
No infection	1.00	1.00
ELIs^†^	0.85 (0.32–2.28)	N/A
Partially hydrolysed formula (pHF)		
No infection	1.00	1.00
Before first month	0.62 (0.16–2.44)	0.23 (0.03–1.95)
Standard formula (SF)		
No infection	1.00	1.00
ELIs^†^	0.96 (0.40–2.42)	0.38 (0.10–1.47)

ITT, intention-to-treat; RR, Relative risk for AD or CMPA; 95% CI, 95% Confidence Interval; ^†^ELIs, Any infection occurring early in life, i.e., before or after the 1st month of life; CMPA + susp, confirmed CMPA and suspected CMPA cases where symptoms improved upon introducing an extensively hydrolyzed formula, but without open food challenge confirmation due to lack of parental consent. For all RR values presented in the table, adjustments were made for country, the mode of the child's delivery, mother's educational level, family history of allergies and urban residence.

The bolded values indicate statistically significant results.

The statistical significance of the results from the ITT analyses with regards to the incidence rates and RR for AD and CMPA for those with and without ELIs were similar to the ones derived from the PP analyses. The results derived from the PP analyses are presented in Supplementary Tables S1–S7.

## Discussion

### Interpreting the impact of ELIs on allergy development

The findings of our current study offer significant insights into how early life infections (ELIs) influence the development of allergic conditions such as Cow's Milk Protein Allergy (CMPA) in infants at high risk for allergies. A particularly notable observation is the protective effect of ELIs against CMPA in the exclusively breastfed (EBF) group. This outcome suggests a complex interplay between the infant's immune system and the anti-inflammatory and other health-protective properties of human milk. These properties appear to synergize with microbial exposures in early life, potentially reducing the risk of allergic diseases in these infants.

This observation aligns with the “hygiene hypothesis” (5, 32), which proposes that certain microbial encounters during early life are crucial for the appropriate maturation of the immune system. According to this hypothesis, these encounters may help the immune system develop a tolerance to various allergens. In the context of EBF, this tolerance may be enhanced due to the unique composition of breast milk, which includes bioactive components known for their immunomodulatory and anti-inflammatory effects.

In line with this, our study observed a trend where the incidence of AD was notably lower in infants with ELIs who were the fed partially hydrolyzed formula (pHF) at 6.5%, compared to those fed the standard formula (SF) at 18.2% (*p* = 0.092), indicating a potentially distinct immune modulation pathway influenced by early dietary factors.

Our study's emphasis on the EBF group is critical, as it highlights the dual role of breast milk in both delivering essential nutrients and acting as a medium for beneficial microbial exposure. The protective effects observed in exclusively breastfed infants underscore the importance of breastfeeding in early life immune modulation and allergy prevention, particularly in infants with a family history of allergies.

The A.R.T. data contributes to the growing understanding of the multifaceted relationship between early life infections, breastfeeding, and allergy development. It underscores the significance of exclusive breastfeeding in shaping infant immune responses and potentially mitigating the risk of allergic diseases like CMPA and AD. These findings support the need for continued research into the complex interactions between diet, microbial exposure, and immune development in early life.

In contrast, the relationship between ELIs and AD appears more nuanced. The observation of lower AD incidence in infants on pHF with post-first month infections suggests that the timing and nature of microbial exposures, coupled with specific feeding practices of infants, may differentially influence the risk of various allergic conditions. This finding opens new avenues for research into the timing of immune system exposures and their long-term impacts on allergy development.

Newborns initially possess an immune system that lacks historical memory and primarily relies on passive immunity. This is facilitated by the transfer of maternal immunoglobulins, specifically IgG and IgA, via the placenta, a process that not only provides critical protection to the newborn but also potentially “primes” specific immune responses (33). Post-birth, breast milk significantly contributes to the neonate's defense, offering bioactive components like immunoglobulins and human milk oligosaccharides (34, 35).

According to ESPGHAN, CMPA is one of the most commonly occurring food allergies in infants and young children, with a prevalence ranging from less than 0.5% to 4.9% (25). Infants diagnosed with CMPA show differences in gut bacteria, both in quantity and dominant species (36). Studies like the KOALA Birth Cohort Study have linked certain microbiome profiles, such as those with an abundance of *Escherichia coli* or *Clostridium difficile*, with increased risks of eczema and other allergic manifestations (37). Therefore, nutritional modulation of the gut microbiome during early life may be crucial in managing allergy risks.

ELIs are responsible for the development of Th1 inflammatory response vs. Th2 allergic trajectory, leading to the hypothesis that repeated infections could cause immune tolerance. Allergic disorders are also influenced by epithelial cells, tissue macrophages, and innate lymphoid cells, all of which can be affected by infections and have early developmental origins, potentially influenced by ELIs, even *in utero* (38, 39).

Neonatal and early life immune interactions are crucial for the development of the immune system, particularly at the respiratory mucosal barrier. Early microbial exposure, including bacterial colonization of the nasal airway and gut, plays a significant role in shaping the immune response and susceptibility to respiratory infections and asthma. As discussed by Gustavo Nino et al. (40), these early microbial-immune interactions contribute to the concept of “innate immune training,” which is the process by which the immune system learns to respond effectively to environmental challenges during critical windows of development (40). Understanding these processes is essential for identifying key pathways and timing for effective immune maturation, which may be modulated by early microbiota and viral exposures.

Research has also shown that breast milk composition, particularly the levels of immune-modulating proteins such as secretory Immunoglobulin A (sIgA) and lactoferrin, can change in response to infant infections. A study by Fatimah et al. (41) demonstrated that exclusive breastfeeding (EBF) is significantly associated with higher levels of sIgA and lactoferrin in toddlers, especially in those with fewer instances of Acute Respiratory Infections (ARIs). Toddlers who were exclusively breastfed with an ARI frequency of less than two times exhibited higher levels of sIgA and lactoferrin compared to those with more frequent ARI episodes (188,901.77 pg/ml vs. 136,683.47 pg/ml for sIgA, and 262.32 ng/ml vs. 181.49 ng/ml for lactoferrin, respectively). This suggests that breast milk adapts to the infant's health status by modulating its immune components in response to infections. Such changes may contribute to enhancing the immune system and potentially reducing allergic diseases in infants.

### Contextualizing findings within the broader research landscape

Our results contribute to a growing body of literature that challenges the notion of a universally protective effect of hygienic practices in early life. They underscore the potential benefits of controlled microbial exposure in infancy, especially in the context of allergy prevention. This perspective is particularly relevant given the increasing prevalence of allergic diseases in industrialized countries, where higher standards of hygiene and reduced microbial diversity are common.

However, the interplay between ELIs, feeding practices, and allergy development is not yet straightforward. Our findings suggest that the protective effect of ELIs against CMPA is particularly pronounced in exclusively breastfed infants. This could be due to the unique composition of breast milk, which not only provides vital nutrients but also shapes the gut microbiota and influences the infant's immune responses.

While the focus of our study was on CMPA and AD, it is also pertinent to consider whether early life infections could influence the development of other allergic diseases, such as food allergies like peanut allergy. Recent studies indicate that the immune-modulating effects of early life infections may not be limited to CMPA and AD. Harald Renz and colleagues (42) have highlighted that early life microbial exposures, including infections, can significantly shape immune system responses, potentially impacting the risk of multiple allergic outcomes beyond the specific conditions we examined (42).

Renz's work emphasizes that disruptions in early immune interactions, often influenced by infections or lack thereof, may alter immune programming, contributing to the development of allergic diseases such as asthma, food allergies, and atopic dermatitis. This suggests that early life infections might offer a broader protective effect across different types of allergies, particularly by promoting a balanced immune response during critical periods of immune system development.

Exploring the effects of early life infections on a wider range of allergic diseases could provide greater insight into whether early microbial exposures influence general immune regulation or if their effects are condition-specific. Such an expanded focus could help establish a more comprehensive understanding of the role of infections in preventing diverse allergic outcomes, contributing to improved allergy prevention strategies.

While controlled microbial exposure in infancy is hypothesized to play a role in allergy prevention, it should be noted that our study does not include direct data on the microbiota. Instead, we propose that early microbial interactions may be beneficial in shaping immune tolerance, potentially reducing the risk of allergic diseases. This hypothesis aligns with current literature, which suggests that a diverse and balanced early microbial environment could contribute to immune system development and allergy prevention. Future studies are needed to collect direct microbiota data and provide more definitive insights into these relationships.

Although microbial exposures during infancy have been linked to immune modulation and potentially reducing allergic outcomes, it is important to emphasize that infection prevention remains a critical component of pediatric healthcare. Breastfeeding, for example, offers dual benefits: not only does it help modulate the immune system to reduce the risk of allergies, but it also provides crucial protection against infectious diseases, which can be life-threatening, particularly in infants at high risk of both infections and allergies. The narrative should therefore reflect the dual role of breastfeeding—supporting both infection prevention and allergy modulation—underscoring that these two objectives are complementary rather than mutually exclusive.

### Implications for pediatric healthcare and allergy prevention

From a clinical perspective, these findings highlight the importance of a balanced approach to infection prevention and management in early life, especially among high-risk infants. Pediatric healthcare providers should consider the potential long-term impact of early microbial exposures on allergy development when advising parents on infection management and hygiene practices.

### Study limitations and areas for further research

While our study offers valuable insights, it is not without limitations. The reliance on parent-reported data for identifying ELIs introduces potential reporting bias, and the multifactorial nature of allergy development complicates the isolation of specific impacts. Exclusively breastfed (EBF) infants have not have been directly exposed to cow's milk protein (CMP). However, exposure to CMP in EBF infants is possible through maternal diets containing dairy, as CMP can pass into breast milk in small amounts. The study's regional focus on three specific countries (Bulgaria, Cyprus, and Greece) may also limit the generalizability of findings to other populations with different cultural, genetic, or environmental characteristics.

Another important limitation is the disproportionately high prevalence of food allergies reported in the family history of atopic comorbidities. Approximately 30% of the families in our cohort reported a history of food allergies, which exceeds the rates typically observed in the literature. This discrepancy may reflect biases in recruitment (e.g., selection of families with greater allergy awareness) or reporting and highlights the need for caution when interpreting the implications of family history data.

Lastly, the relatively short follow-up period (six months) restricts conclusions about the long-term impact of ELIs and feeding practices on allergy development. A key limitation that merits attention, particularly in contextualizing the findings of “borderline significance,” is the relatively small sample size, with only 17/4 subjects having AD/CMPA, respectively, with ELIs.

Future studies could address these limitations by incorporating more objective measures of ELIs and by including more diverse and wider populations.

## Conclusion

In conclusion, our findings highlight a potentially protective role of ELIs against the development of CMPA, particularly in exclusively breastfed infants, and suggest a trend towards lower AD incidence in infants fed with pHF. No significant impact of ELIs was observed for AD. These results underscore the complex relationship between early microbial exposures, feeding practices, and immune development, thus paving the way for further research in this crucial area of pediatric health.

## Data Availability

The original contributions presented in the study are included in the article/Supplementary Material, further inquiries can be directed to the corresponding author.
