# Supplementary material for: Early life acute infections and risk for cow's milk protein allergy or atopic dermatitis at 6 months of age in high risk for allergy infants
Source: Front Pediatr. 2024 Dec 16;12:1424331. doi: 10.3389/fped.2024.1424331 (PMC11697985; doi:10.3389/fped.2024.1424331)
Supplement: Supplementary file 2 [file Datasheet2.docx]

**Supplementary Table 4.** Descriptive characteristics of study participants allocated to the three treatment arms at baseline (**PP dataset**).

|  | **EBF group**  **(N=161)** | **pHF group**  **(N=105)** | **SF group**  **(N=120)** | **p-value** | **Total sample**  **(N=386)** |
| --- | --- | --- | --- | --- | --- |
| **Infant’s characteristics** |  |  |  |  |  |
| Country of infant’s birth |  |  |  |  |  |
| Bulgaria, n (%) | 35 (21.7)^a,b^ | 45 (42.9)^a^ | 58 (48.3)^b^ | **<0.001** | 138 (35.8) |
| Cyprus, n (%) | 33 (20.5)^a,b^ | 45 (42.9)^a^ | 43 (35.8)^b^ |  | 121 (31.3) |
| Greece, n (%) | 93 (57.8)^a,b^ | 15 (14.3)^a^ | 19 (15.8)^b^ |  | 127 (32.9) |
| Birth weight, g, mean (SD) | 3290.1 (374.3) | 3257.1 (431.4) | 3246.7 (449.6) | 0.656 | 3267.6 (413.9) |
| Length at baseline, cm, mean (SD) | 50.2 (2.0)^a^ | 49.5 (1.8)^a^ | 49.8 (2.0) | **0.029** | 49.9 (2.0) |
| Gender, female, n (%) | 77 (47.8) | 47 (44.8) | 55 (45.8) | 0.878 | 179 (46.4) |
| **Mother’s characteristics** |  |  |  |  |  |
| Age, years, mean (SD) | 32.9 (4.7)^a,b^ | 31.3 (4.8)^a^ | 31.4 (5.4)^b^ | **0.010** | 32.0 (5.0) |
| Educational level |  |  |  |  |  |
| ≤ 14 years, n (%) | 44 (27.3)^b^ | 43 (41.0) | 52 (43.3)^b^ | **0.010** | 139 (36.0) |
| >14 years, n (%) | 117 (72.7)^b^ | 62 (59.0) | 68 (56.7)^b^ |  | 247 (64.0) |
| Mother smoking during pregnancy, n (%) | 10 (6.2)^b^ | 10 (9.5) | 18 (15.0)^b^ | **0.050** | 38 (9.8) |
| **Family characteristics** |  |  |  |  |  |
| Urban residence, n (%) | 150 (93.2) | 96 (91.4) | 102 (85.7) | 0.103 | 348 (90.4) |
| Presence of pets indoors at home, n (%) | 29 (18.0) | 25 (23.8) | 28 (23.3) | 0.699 | 82 (21.2) |
| **Medical history** |  |  |  |  |  |
| Family history of: |  |  |  |  |  |
| Allergic asthma, n (%) | 53 (32.9) | 25 (23.8) | 30 (25.0) | 0.184 | 108 (28.0) |
| Rhinitis, n (%) | 96 (59.6) | 52 (49.5) | 59 (49.2) | 0.135 | 207 (53.6) |
| Atopic dermatitis, n (%) | 59 (36.6) | 26 (24.8) | 34 (28.3) | 0.095 | 119 (30.8) |
| Urticaria, n (%) | 28 (17.4) | 15 (14.3) | 21 (17.5) | 0.760 | 64 (16.6) |
| Food allergy, n (%) | 47 (29.2) | 31 (29.5) | 33 (27.5) | 0.934 | 111 (28.8) |
| Occurrence of early life infections in infants |  |  |  |  |  |
| No infections, n (%) | 126 (78.3)^a^ | 63 (60.0)^a^ | 79 (65.8) | **0.005** | 368 (69.4) |
| Before 1^st^ month, n (%) | 6 (3.7) | 2 (1.9) | 6 (5.0) |  | 14 (3.6) |
| After 1^st^ month, n (%) | 29 (18.0)^a^ | 40 (38.1)^a^ | 35 (29.2) |  | 104 (26.9) |

**EBF:** exclusive breastfeeding; **pHF:** partially hydrolysed formula; **SF:** Standard formula; **PP:** Per-Protocol; **N:** Number of study participants; **n:** number of non-missing observations; **SD:** Standard Deviation; **IQR:** Interquartile Range.

p-values for the comparison of categorical variables derived from the chi-square test or the Fisher exact test, whenever appropriate. P-values for the comparison of continuous variables derived from one-way ANOVA or the Kruskal Wallis test for normally and non-normally distributed variables respectively. All p-values in bold indicate statistically significant differences among treatment arms. Percentages sharing the same superscript letter within the same raw are statistically significantly different between them, according to pairwise comparisons using the Bonferroni correction to account for type I error.

**Supplementary Table 5:** Incidence of atopic dermatitis (AD) or cow’s milk protein allergy (CMPA) at the age of 6 months for infants with or no infections at early life, in the total sample and by study group (**PP dataset**).

|  | **Atopic dermatitis (AD)** | | | **Cow’s milk protein allergy (CMPA +susp)** | | |
| --- | --- | --- | --- | --- | --- | --- |
| **Total sample** | | | | | | |
| **Timing of infections** | **No AD** | **AD** | ***p*-value** | **No CMPA** | **CMPA** | ***p*-value** |
| No infection | 211 (67.4%) | 57 (78.1%) | 0.204 | 231 (67.0%)^a^ | 37 (90.2%)^a^ | **0.008** |
| Before first month | 12 (3.8%) | 2 (2.7%) |  | 14 (4.1%) | 0 (0.0%) |  |
| After first month | 90 (28.8%) | 14 (19.2%) |  | 100 (29.0%)^a^ | 4 (9.8%)^a^ |  |
| Total | 313 (100.0%) | 73 (100.0%) |  | 345 (100.0%) | 41 (100.0%) |  |
| **Exclusive Breastfeeding (EBF)** | | | | | | |
| No infection | 99 (76.7%) | 27 (84.4%) | 0.405 | 106 (75.2%) | 20 (100.0%) | **0.042** |
| Before first month | 6 (4.7%) | 0 (0.0%) |  | 6 (4.3%) | 0 (0.0%) |  |
| After first month | 24 (18.6%) | 5 (15.6%) |  | 29 (20.6%) | 0 (0.0%) |  |
| Total | 129 (100.0%) | 32 (100.0%) |  | 141 (100.0%) | 20 (100.0%) |  |
| **Partially hydrolysed formula (pHF)** | | | | | | |
| No infection | 54 (58.1%) | 9 (75.0%) | 0.079 | 57 (58.2%) | 6 (85.7%) | 0.352 |
| Before first month | 1 (1.1%) | 1 (8.3%) |  | 2 (2.0%) | 0 (0.0%) |  |
| After first month | 38 (40.9%) | 2 (16.7%) |  | 39 (39.8%) | 1 (14.3%) |  |
| Total | 93 (100.0%) | 12 (100.0%) |  | 98 (100.0%) | 7 (100.0%) |  |
| **Standard formula (SF)** | | | | | | |
| No infection | 58 (63.7%) | 21 (72.4%) | 0.681 | 68 (64.2%) | 11 (78.6%) | 0.470 |
| Before first month | 5 (5.5%) | 1 (3.4%) |  | 6 (5.7%) | 0 (0.0%) |  |
| After first month | 28 (30.8%) | 7 (24.1%) |  | 32 (30.2%) | 3 (21.4%) |  |
| 0.303Total | 91 (100.0%) | 29 (100.0%) |  | 106 (100.0%) | 14 (100.0%) |  |

**PP**: Per-Protocol; P-values for the comparison of categorical variables derived from the chi-square test or the Fisher exact test, whenever appropriate. All P-values in bold indicate statistically significant differences among treatment arms; Percentages sharing the same superscript letter (i.e., a) within the same raw are statistically significantly different between them, according to pairwise comparisons using the Bonferroni correction to account for type I error

**Supplementary Table 6**: Incidence of atopic dermatitis (AD) or cow’s milk protein allergy (CMPA) at the age of 6 months for infants with or no infections at early life, in the total sample and by study group (**PP dataset)**.

| **Timing of infections** | | | | | | | | | |
| --- | --- | --- | --- | --- | --- | --- | --- | --- | --- |
| **Total sample** | | | | | | | | | |
| **Atopic dermatitis (AD)** | **No infection** | **Before first month** | **After first month** | ***p*-value** | **Cow’s milk protein allergy (CMPA +susp)** | **No infection** | **Before first month** | **After first month** | ***p*-value** |
| No AD | 211 (78.7%) | 12 (85.7%) | 90 (86.5%) | 0.204 | No CMPA | 231 (86.2%)* | 14 (100.0%) | 100 (96.2%*) | **0.008** |
| AD | 57 (21.3%) | 2 (14.3%) | 14 (13.5%) |  | CMPA | 37 (13.8%)* | 0 (0.0%) | 4 (3.8%)* |  |
| Total | 268 (100.0%) | 14 (100.0%) | 104 (100.0%) |  | Total | 268 (100.0%) | 14 (100.0%) | 104 (100.0%) |  |
| **Exclusive Breastfeeding (EBF)** | | | | | | | | | |
| No AD | 99 (78.6%) | 6 (100.0%) | 24 (72.8%) | 0.405 | No CMPA | 106 (84.1%) | 6 (100.0%) | 29 (100.0%) | **0.042** |
| AD | 27 (21.4%) | 0 (0.0%) | 5 (17.2%) |  | CMPA | 20 (15.9%) | 0 (0.0%) | 0 (0.0%) |  |
| Total | 126 (100.0%) | 6 (100.0%) | 29 (100.0%) |  | Total | 126 (100.0%) | 6 (100.0%) | 29 (100.0%) |  |
| **Partially hydrolysed formula (pHF)** | | | | | | | | | |
| No AD | 54 (85.7%) | 1 (50.0%) | 38 (95.0%) | 0.079 | No CMPA | 57 (90.5%) | 2 (100.0%) | 39 (97.5%) | 0.352 |
| AD | 9 (14.3%) | 1 (50.0%) | 2 (5.0%) |  | CMPA | 6 (9.5%) | 0 (0.0%) | 1 (2.5%) |  |
| Total | 63 (100.0%) | 2 (100.0%) | 40 (100.0%) |  | Total | 63 (100.0%) | 2 (100.0%) | 40 (100.0%) |  |
| **Standard formula (SF)** | | | | | | | | | |
| No AD | 58 (73.4%) | 5 (83.3%) | 28 (80.0%) | 0.681 | No CMPA | 68 (86.1%) | 6 (100.0%) | 32 (91.4%) | 0.470 |
| AD | 21 (26.6%) | 1 (16.7%) | 7 (20.0%) |  | CMPA | 11 (13.9%) | 0 (0.0%) | 3 (8.6%) |  |
| Total | 79 (100.0%) | 6 (100.0%) | 35 (100.0%) |  | Total | 79 (100.0%) | 6 (100.0%) | 35 (100.0%) |  |

**PP**: Per protocol; P-values for the comparison of categorical variables derived from the chi-square test or the Fisher exact test, whenever appropriate. All P-values in bold indicate statistically significant differences among treatment arms; Percentages sharing the same superscript letter (i.e., a) within the same raw are statistically significantly different between them, according to pairwise comparisons using the Bonferroni correction to account for type I error

**Supplementary Table 7**: Relative risks for atopic dermatitis (AD) or cow’s milk protein allergy (CMPA) at the age of 6 months based on the presences or not of early life infection, in the total sample and by study group (**PP dataset**).

|  | **Atopic dermatitis (AD)** | **Cow’s milk protein allergy (CMPA +susp)** |
| --- | --- | --- |
| **Total sample** |  |  |
| **Timing of infections** | **RR (95% CI)** | **RR (95% CI)** |
| No infection | 1.00 | 1.00 |
| Before first month | 0.67 (0.18-2.47) | N/A |
| After first month | 0.63 (0.37-1.09) | **0.28 (0.10-0.76)** |
| **Exclusive Breastfeeding (EBF)** |  |  |
| No infection | 1.00 | 1.00 |
| Before first month | Ν/Α | N/A |
| After first month | 0.81 (0.34-1.91) | N/A |
| **Partially hydrolysed formula (pHF)** |  |  |
| No infection | 1.00 | 1.00 |
| Before first month | 3.50 (0.77-15.88) | N/A |
| After first month | 0.35 (0.08 -1.54) | 0.26 (0.03-2.10) |
| **Standard formula (SF)** |  |  |
| No infection | 1.00 | 1.00 |
| Before first month | 0.63 (0.10-3.89) | N/A |
| After first month | 0.75 (0.35-1.60) | 0.62 (0.18-2.07) |

**PP**: Per Protocol; **RR**: Relative risk for AD or CMPA
