# Supplementary material for: Early life acute infections and risk for cow's milk protein allergy or atopic dermatitis at 6 months of age in high risk for allergy infants
Source: Front Pediatr. 2024 Dec 16;12:1424331. doi: 10.3389/fped.2024.1424331 (PMC11697985; doi:10.3389/fped.2024.1424331)
Supplement: Supplementary file 3 [file Datasheet3.doc]

**Supplementary Table 8**: Cumulative incidence of atopic dermatitis (AD) or cow’s milk protein allergy (CMPA) at the age of 6 months for infants with or no infections at early life, in the total sample and by study group (**ITT analyses)**.

| **Presence of infections** | | | | | | | | |
| --- | --- | --- | --- | --- | --- | --- | --- | --- |
| **Total sample** | | | | | | | | |
| **Atopic dermatitis**  **(AD)** | **No infection** | **ELIs†** | ***p*-value** | **Cow’s milk protein allergy**  **(CMPA +susp)** | **No infection** | **ELIs†** | ***p*-value** |  |
| No AD | 347 (83.2%) | 117 (87.3%) | 0.258 | No CMPA | 265 (86.6%)^a^ | 128 (97.0%)^a^ | **0.001** |  |
| AD | 70 (16.8%) | 17 (12.7%) |  | CMPA | 41 (13.4%)^a^ | 4 (3.0%)^a^ |  |  |
| Total | 417 (100.0%) | 134 (100.0%) |  | Total | 306 (100.0%) | 132 (100.0%) |  |  |
| **Exclusive Breastfeeding (EBF)** | | | | | | | | |
| No AD | 144 (81.8%) | 38 (86.4%) | 0.476 | No CMPA | 125 (82.6%) | 44 (100.0%) | **0.009** |  |
| AD | 32 (18.2%) | 6 (13.6%) |  | CMPA | 20 (13.8%) | 0 (0.0%) |  |  |
| Total | 176 (100.0%) | 44 (100.0%) |  | Total | 145 (100.0%)  P=0.313  **P=0.080** | 44 (100.0%) |  |  |
| **Partially hydrolysed formula (pHF)**  P=0.264  P=0.314 | | | | | | | | |
| No AD | 100 (87.7%) | 43 (93.5%) | 0.285 | No CMPA | 65 (90.3%) | 43 (97.2%) | 0.124 |  |
| AD | 14 (12.3%) | 3 (6.5%) |  | CMPA | 7 (9.7%) | 1 (2.3%) |  |  |
| Total | 114 (100.0%)  P=0.557 | 46 (100.0%) |  | Total | 72 (100.0%) | 44 (100.0%) |  |  |
| **Standard formula (SF)**  **P=0.092** | | | | | | | | |
| No AD | 103 (81.1%) | 36 (81.8%) | 0.916 | No CMPA | 75 (84.3%) | 41 (93.2%) | 0.148 |  |
| AD | 24 (18.9%) | 8 (18.2%) |  | CMPA | 14 (15.7%) | 3 (6.8%) |  |  |
| Total | 127 (100.0%) | 44 (100.0%) |  | Total | 89 (100.0%) | 44 (100.0%) |  |  |

**†ELIs:** Any infection occurring early in life, i.e., before or after the 1^st^ month of life; **ITT:** intention-to-treat; P-values for the comparison of categorical variables derived from the chi-square test or the Fisher exact test, wherever appropriate. All P-values in bold font indicate statistically significant differences among study groups; Percentages sharing the same superscript letter within the same line are statistically significantly different between groups.
